# Fellows’ Hypophosphites

**Published:** 1882-02-20

**Authors:** 


THE advertisement of Fellows' Hypophosphites should be carefully examined. It is an excellent preparation, possessing stimulant, tonic and nutritive properties of a high order—especially adapted to consumptives and low nervous states of the system.

